# Sewers and Their Evils

**Published:** 1865-12

**Authors:** 


### SEWERS AND THEIR EVILS.

The immense extent of the London system of sewerage probably converts the sewers into one enormous cesspool. It was, of course, the decomposition of the animal excrements which gave rise to the dangerous vapors issuing from the cesspools. Now, if these excrements are allowed, in consequence of the length of the sewers through which they now have to pass, to decompose, as they decomposed in the ancient cesspools, why should not the vapors and gases arising from the decomposition in the sewers produce as noxious effects as they produced when they escaped from the cesspools? We, some years ago, suggested this question, Whether our present system of sewage would not become one enormous cesspool; and whether some special provision ought not to be made for the escape, by high shafts, or neutralization of the products of decomposition? If it be true that the contents of our sewers in London undergo decomposition just as they underwent decomposition in the old cesspools, surely it was something akin to madness to set loose all the products of the decomposition at our very doors and under our very noses. But all this matter rexuires investigation; and interesting would it be, if we could get some sure information as to the ordinary health of those men who pass many hours in these sewers, and whom we occasionally see emerging from iron traps, with lantern and heavy jack-boots. What effect does the inhaling of the vapors of sewers have upon them? Perhaps some of our readers can tell us something of this; and we may add, that we wish Dr. Fuller had furnished the *Times* with some positive proof that the issue of gases from sewers had injured human constitutions and produced diseases.

Dr. Miller, Professor of Chemistry in King's College, says truly enough, that sewers must be ventilated—*i. e.* the gases must be let out of them—so long as it is necessary for men to pass through them; and he recommends the process of ventilation and disinfection proposed by Dr. Stenhouse.

It consists in suspending charcoal in the ventilating openings. In London, the plan has been carried out by the engineer to the Commissioners of Sewers, with the sanction of Dr. Letheby, and both these gentlemen have reported strongly in its favor. There is placed in each ventilating opening a box, within which are three or four perforated shelves, and on each side of these shelves is a layer of wood charcoal; openings are made at the top and bottom of the box, to allow the free passage of the air; the whole of the air which escapes from the sewer is obliged to pass through the box and over the charcoal before it reaches the outer atmosphere. The offensive and noxious gases are speedily absorbed by the charcoal, and are oxidized within its pores, by which means they are converted into a harmless substance, destitute of odor. "The method is so simple and effectual," says Dr. Miller, "that it ought at once to be put in practice, while yet there is time." * * * *

Dr. Herbert Barker, who has proved himself to be a high authority on the subject of disinfection, speaks of ozone as being "Nature's grand atmospheric disinfectant." His observations are of much interest, and the practical conclusions recommended worthy of consideration, especially in reference to this matter of the cholera. We conclude that Dr. Barker has satisfactory proofs of the fact that ozone is really absent in the district where cholera rages, &c. Of course, the full establishment of this fact is very important.

"In the neighborhood of cesspools, all evidence of the presence of ordinary atmospheric ozone is lost. When ozone is abundant in the air, it may be detected on the windward side of a stable, or cowshed, or manure-heap, but not on the leeward side. It may be observed abundantly immediately on the windward side of a town, and not a trace of it discovered at the same time on the leeward side. The ozone test paper, in an ill-ventilated church, when full of persons, will give no reaction. I have evidence from my own experience that the diffusion of ozonized air through the apartments of persons suffering from fevers, is of immense service, in that it keeps the room free of oppression, and effectually destroys the offensive odors arising from the gaseous excreta of the subject. Ozone, in its action as a deodorizer, closely resembles chlorine. It can be employed permanently by a single process with ventilation. Ozone may be prepared by Siemen's cylinder, the air driven through the cylinder being ozonized by sparks from Ruhmkorf's coil. This method can be adopted only in hospitals, as skilled hands are required for its management. Fortunately, we have a means of generating ozone from phosphorus, which is ready for use at any moment, and with little trouble. Two sticks of phosphorus, each two inches in length, made very clean by scraping, if covered with oxide, and half covered with water, will yield in an hour sufficient ozone, in a room of 3,000 cubic feet, to be detectable by Schönbein's test in every part, and this even when there is good ventilation. The objection to the production of ozone, that there is not a sufficient bulk of water to absorb the fumes of phosphoric acid, may be obviated by using a vessel containing a larger quantity of water, and by floating the phosphorus at the proper depth upon its surface. The degree of evolution of ozone may be tested by a slip of Schönbein's paper. It is very remarkable that, during the prevalence of cholera in any district, ozone has been observed to be absent in that district; not the smallest trace has been discoverable by the test papers."—*Medical News & Library*, from *British Medical Journal.*

